# Secretory breast carcinoma: morphologic and molecular heterogeneity with indicators of aggressive potential in a cohort of 29 cases

**DOI:** 10.1002/2056-4538.70060

**Published:** 2025-11-06

**Authors:** Huayan Ren, Wanting Tong, Jiayue Ma, Xinyan Chen, Huifen Huang, Na Wei, Yuqiong Liu, Minglei Yang, Lan Zhang, Huixiang Li

**Affiliations:** ^1^ Department of Pathology The First Affiliated Hospital of Zhengzhou University Zhengzhou PR China; ^2^ College of Basic Medical Sciences Zhengzhou University Zhengzhou PR China; ^3^ Hillman Cancer Center University of Pittsburgh Medical Center Pittsburgh PA USA; ^4^ Department of Biological Sciences University of Pittsburgh Pittsburgh PA USA

**Keywords:** secretory breast carcinoma, *ETV6*‐*NTRK3* fusion, *NTRK3* rearrangement, pan‐TRK, immunohistochemistry, fluorescence *in situ* hybridisation (FISH), RNA‐seq, DNA‐seq

## Abstract

Secretory breast carcinoma (SBC) is a rare tumour defined by *ETV6‐NTRK3* rearrangement, but its clinicopathological spectrum and potential for aggressive behaviour remain incompletely characterised. We retrospectively reviewed 29 SBCs diagnosed between 2014 and 2024, including 28 females and one male aged 12–63 years (median 44). Twenty‐eight tumours arose in the breast parenchyma and one in axillary accessory breast tissue. Histologically, microcystic and tubular patterns predominated and carcinoma *in situ* was common. Most tumours were nuclear grade 1 with rare mitoses (0–1/10 high‐power fields, HPF). A single patient with distant metastasis harboured a solid‐predominant tumour showing nuclear grade 2–3, brisk mitoses (6/10 HPF), and multifocal necrosis. All tumours demonstrated diffuse S100 and pan‐TRK expression. Oestrogen and/or progesterone receptor staining was observed in 16 of 29 cases (2–30% of tumour cells), and all were HER2 negative or low (0–1+). Ki‐67 ranged from 3% to 20% (mean 7%). Fluorescence *in situ* hybridisation (FISH) was positive in 17 of 17 tested tumours (14 *ETV6‐NTRK3* dual‐fusion; 3 *NTRK3* break‐apart). In the metastatic case, RNA sequencing confirmed canonical *ETV6‐NTRK3* fusion, while targeted DNA sequencing identified additional variants of uncertain significance (VUS) – *RANBP2* p.S1843R, *NUP107* p.K382Q, *NCOR1* p.A1947V (missense), and *PREX2* p.G606G (synonymous). All patients underwent surgery, and 14 received adjuvant chemotherapy. During follow‐up ranging from 6 to 135 months (median 76), one patient developed lung metastasis and was alive with disease at 88 months; the remaining 28 patients were alive without recurrence or metastasis. In summary, SBC is typically indolent and characterised by *ETV6‐NTRK3* rearrangement with diffuse pan‐TRK/S100 positivity. A solid‐predominant pattern with increased cytological atypia, mitotic activity, and necrosis may indicate aggressive potential. Routine *NTRK* testing supports diagnosis and may help identify patients who could benefit from TRK‐inhibitor therapy in advanced disease.

## Introduction

Secretory breast carcinoma (SBC) is a rare and distinct subtype of invasive breast carcinoma, initially described in children and adolescents [1]. Subsequent studies, however, have demonstrated that SBC can arise across a wide age spectrum and has also been reported in male patients [2, 3]. Histologically, the classic features comprise microcystic structures with variable eosinophilic or basophilic secretions within and outside the tumour cells, often accompanied by stromal hyalinisation and an *in situ* carcinoma component of variable extent [3]. Immunohistochemically, SBC typically shows diffuse S100 positivity. At the molecular level, the recurrent *ETV6–NTRK3* gene fusion is regarded as a diagnostic hallmark [4, 5]. Most tumours display a triple‐negative phenotype for ER, PR, and HER2, although ER and/or PR positivity has been documented in a minority of cases [6].

Although SBC is generally considered indolent with a favourable prognosis, regional lymph node and distant metastases have occasionally been reported, suggesting that a subset of patients may follow a more aggressive clinical course [7, 8, 9, 10]. In this study, we analysed 29 cases of SBC diagnosed between 2014 and 2024 at the Department of Pathology, the First Affiliated Hospital of Zhengzhou University, with the aim of characterising their clinicopathological, immunophenotypic, and molecular features, and of exploring potential prognostic factors to better inform pathological diagnosis and clinical management.

## Materials and methods

### Ethics statement

The study protocol was reviewed and approved by the Ethics Committee of the First Affiliated Hospital of Zhengzhou University. Written informed consent was obtained from all participants or their legal guardians.

### Clinical data

Twenty‐nine cases of secretory breast carcinoma diagnosed between 2014 and 2024 were retrieved from the Department of Pathology, the First Affiliated Hospital of Zhengzhou University. All cases were independently reviewed and confirmed by two breast pathologists. Clinical information and follow‐up data were obtained from the hospital electronic medical record system and supplemented by telephone interviews.

### Immunohistochemistry

All specimens were fixed in 3.7% neutral buffered formalin, routinely processed, paraffin embedded, and cut into 4‐μm sections for HE staining. Immunohistochemistry was performed on an automated immunostainer (Roche, Indianapolis, IN, USA). Primary antibodies included oestrogen receptor (ER, clone 1D5; Dako, Denmark), progesterone receptor (PR, clone PGR636; Dako), S100 (polyclonal; Dako), HER2 (clone 4B5; Roche), pan‐TRK (clone EPR17341; Roche), and Ki‐67 (clone GM027; Gene Tech, Shanghai, PR China). Most immunohistochemical stains were performed at the time of the original diagnoses; pan‐TRK was not performed for cases diagnosed before 2022 and was subsequently carried out retrospectively on archived material for this study. All antibodies were applied according to the manufacturers' instructions using the standard protocol of the automated platform. ER and PR were evaluated according to the proportion of positive tumour cell nuclei. S100 was assessed as diffuse nuclear and cytoplasmic staining. Pan‐TRK was interpreted based on nuclear positivity of tumour cells. HER2 evaluation was based on the 2023 American Society of Clinical Oncology/College of American Pathologists guideline update [11].

### Fluorescence *in situ* hybridisation (FISH)

*ETV6‐NTRK3* fusion (t[12;15]) probes and *NTRK1/NTRK2/NTRK3* break‐apart probe kits (Anbiping, Guangzhou, PR China) were used following the manufacturers' instructions. At least 50 tumour cells were evaluated. For break‐apart probes, separation of red and green signals in >15% of cells was interpreted as positive for *NTRK*1/2/3 rearrangement [12]. For *ETV6‐NTRK3* dual‐fusion probes, <10% of fused signals was considered negative, >50% was positive, and 10–50% was equivocal, requiring confirmation with break‐apart probes [13].

### RNA sequencing (RNA‐seq)

The sequencing libraries were constructed using the IGT® Fast Stranded RNA Library Prep Kit v2.0 (iGeneTech, Beijing, PR China). Total RNA was fragmented and reverse‐transcribed into first‐strand cDNA with random primers, followed by second‐strand synthesis. The resulting double‐stranded cDNA underwent end repair, 3′ A‐tailing, and adapter ligation. After 15 cycles of PCR amplification with unique dual indexes, PCR products were purified with IGT® Pure Beads (iGeneTech). Libraries were further enriched with biotinylated probes targeting 554 genes, using the TargetSeq One® Hyb and Wash Kit v2.0 (iGeneTech) according to the manufacturer's instructions. The final enriched libraries were sequenced on the Illumina NextSeq 550 platform to generate 2 × 150 bp paired‐end reads. The raw sequencing data were converted to FASTQ files using the Bcl2fastq tool, followed by quality control and filtering with fastp. The filtered FASTQ files were then aligned to the human reference genome using STAR‐Fusion software (v1.14.0; https://github.com/STAR-Fusion/STAR-Fusion/wiki) for fusion transcript analysis.

### DNA sequencing (DNA‐seq)

A sample of 200 ng genomic DNA of each sample was interrupted, repaired, and affixed with an Adapter (IGT® Enzyme Plus Library Prep Kit V3, iGeneTech). After library construction, the exons of 794 genes were captured with a designed targeted sequencing panel using the TargetSeq One® Hyb & Wash Kit v2.0 (iGeneTech) and sequenced on the Illumina NextSeq 550Dx platform (Illumina, Inc., San Diego, CA, USA) to generate 150‐bp paired‐end reads. Raw reads were filtered to remove low‐quality reads and adapter‐contaminated reads using fastp. Clean reads were aligned to the human reference genome GRCh37 using Burrows–Wheeler Aligner Maximal Exact Matches (BWA‐MEM, v0.7.17) [14]. PCR duplicates were removed, and single‐nucleotide variants (SNVs) and insertions/deletions (indels) were called and annotated using Mutect2 wrapped in Genome Analysis Toolkit (GATK, v4.2.0.0) [15].

## Results

### Clinical features

The cohort comprised 29 patients with secretory breast carcinoma (SBC), including 28 females and 1 male. The age at diagnosis ranged from 12 to 63 years (mean, 42 years; median, 44 years). Twenty‐three patients presented with a palpable, firm, and mobile breast mass, while six tumours were detected incidentally on imaging. Four patients experienced pain, whereas 25 were asymptomatic. Tumours were located in the right breast in 19 cases and in the left breast in 10 cases. Ultrasonography (21 cases) typically revealed hypoechoic or mixed‐echogenicity masses with relatively well‐circumscribed margins. Twenty‐eight tumours arose within the breast parenchyma, and one was located in the axilla (Case 23), where a small amount of adjacent breast tissue was also identified histologically. A detailed summary of clinicopathologic characteristics is provided in Table 1.

**Table 1:** Clinicopathologic features, immunohistochemical profile, molecular findings, therapies, and follow‐up data of 29 cases of breast secretory carcinoma

Case no.	Sex	Age (year)	Site	Size (cm)	Histological architecture	Nuclear grade	Mitoses (10 HPF)	IHC						*NTRK3* gene rearrangement	Therapy	Follow‐up (months)
								ER	PR	HER2	Ki67	S100	Pan‐TRK			
1	F	48	L	4.5	90% MC + 10% T	1	1	−	−	1+	15%	+	+	ND	MRM + CT	135
2	F	51	R	0.5	80% MC + 20% T	1	0	5%+	2%+	1+	5%	+	+	FISH, Positive	SM + CT	133
3	M	12	L	1.6	80% MC + 10%T + 10% P	1	1	−	−	0	10%	+	+	ND	SM	126
4	F	36	L	1.1	70% MC + 20%T + 10% P	1	0	−	−	0	5%	+	+	ND	BCS	123
5	F	34	R	1.5	70% MC + 15%T + 15% P	1	0	−	−	0	3%	+	+	FISH, Positive	BCS + CT	111
6	F	54	L	0.6	95% MC + 5% T	1–2	0	−	−	0	5%	+	+	ND	SM	104
7	F	63	R	1.1	90% MC + 10% P	1	0	−	−	1+	5%	+	+	ND	SM	100
8	F	42	R	0.8	85% MC + 15% T	1	0	−	−	0	5%	+	+	FISH, Positive	BCS	96
9	F	35	R	1.1	90% MC + 10% T	1	1	10%+	−	0	10%	+	+	FISH, Positive	BCS	91
10	F	29	R	1.4	90% MC + 5%T + 5% P	1	0	3%+	−	0	5%	+	+	ND	BCS + CT	89
11	F	47	R	1.0	**95% S + 5% MC**	**2–3**	6	2%+	−	0	20%	+	+	FISH/NGS, Positive	MRM + CT	**88, Lung metastasis**
12	F	34	R	1.5	90% MC + 10% T	1	1	20%+	−	0	10%	+	+	FISH, Positive	BCS	88
13	F	44	R	1.3	80% MC + 10%T + 10% P	1	0	30%+	−	0	10%	+	+	FISH, Positive	SM + CT	85
14	F	51	R	1.2	85% MC + 10%T + 5% P	1	0	−	−	0	10%	+	+	ND	SM + CT	79
15	F	57	R	1.5	**90% P + 5%MC + 5% T**	1–2	0	−	−	0	10%	+	+	FISH, Positive	MRM + CT	76
16	F	53	L	1.2	80% MC + 10%T + 10% S	1	2	20%+	30%+	0	10%	+	+	FISH, Positive	BCS	75
17	F	28	L	1.3	90% MC + 10% T	1	0	3%+	−	0	5%	+	+	FISH, Positive	BCS	67
18	F	35	R	1.7	90% MC + 5%T + 5% S	1–2	1	−	−	0	15%	+	+	ND	BCS + CT	56
19	F	48	R	1.5	70% MC + 20%P + 10% T	1	0	−	−	0	5%	+	+	ND	MRM + CT	43
20	F	45	L	1.2	90% MC + 10% T	1	0	−	−	0	5%	+	+	FISH, Positive	SM	40
21	F	46	R	1.0	95% MC + 5% T	1	0	5%+	−	0	5%	+	+	FISH, Positive	MRM + CT	38
22	F	45	R	1.4	80% MC + 10%T + 10% P	1	1	10%+	10%+	1+	5%	+	+	FISH, Positive	BCS + CT	38
23	F	31	R	0.9	90% MC + 5%T + 5% P	1	0	5%+	−	1+	3%	+	+	ND	BCS	37
24	F	54	L	1.7	95% MC + 5% T	1	1	20%+	−	1+	5%	+	+	ND	SM + CT	30
25	F	28	R	1.5	90% MC + 5%T + 5% S	1–2	0	10%+	10%+	1+	10%	+	+	FISH, Positive	BCS	29
26	F	32	R	1.3	95% MC + 5% T	1	0	20%+	−	1+	3%	+	+	FISH, Positive	BCS	18
27	F	34	R	1.3	85% MC + 10%T + 5% P	1–2	1	30%+	−	1+	10%	+	+	FISH, Positive	SM	12
28	F	48	L	0.9	90% MC + 5%T + 5% P	1	0	10%+	−	0	5%	+	+	FISH, Positive	SM + CT	8
29	F	53	L	1.1	95% MC + 5% T	1	0	−	−	1+	5%	+	+	ND	BCS	6

BCS, breast conserving surgery; CT, chemotherapy; F, female; FISH, fluorescence *in situ* hybridization; HPF, high‐power fields; L, left breast; M, male; MC, microcystic; MRM, modified radical mastectomy; ND, not done; NGS, next‐generation sequencing; P, papillary; R, right breast; S, solid; SM, simple mastectomy; T, tubular. Bold text identifies the only case with distant metastasis (case 11) and the case with a predominantly papillary architecture (case 15).

### Histopathological features

**Figure 1:**
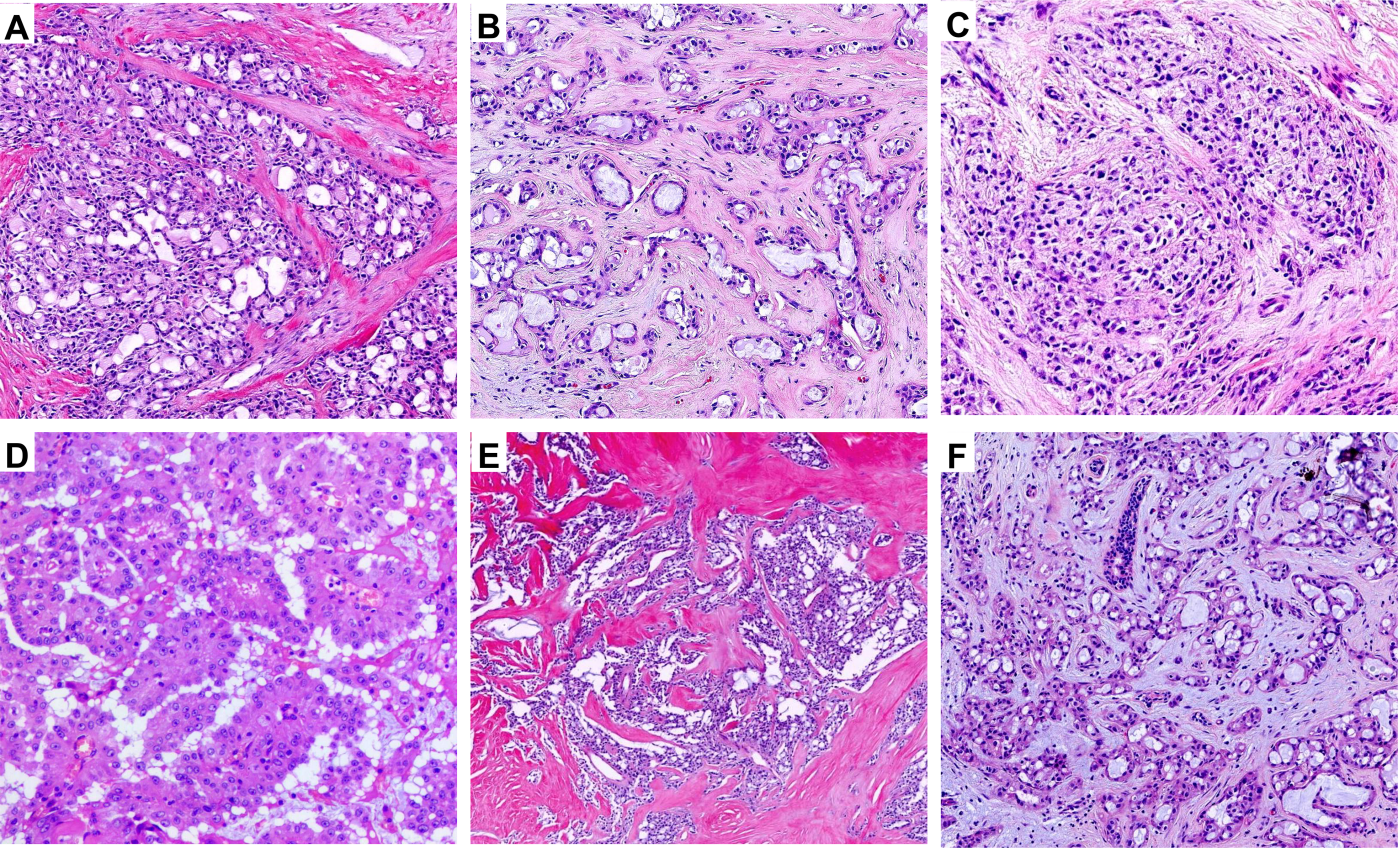
Representative histological patterns of breast secretory carcinoma. (A) Microcystic, (B) tubular, (C) solid, and (D) papillary architectures. (E) Hyalinised/sclerotic stroma. (F) Myxoid stromal change.

**Figure 2:**
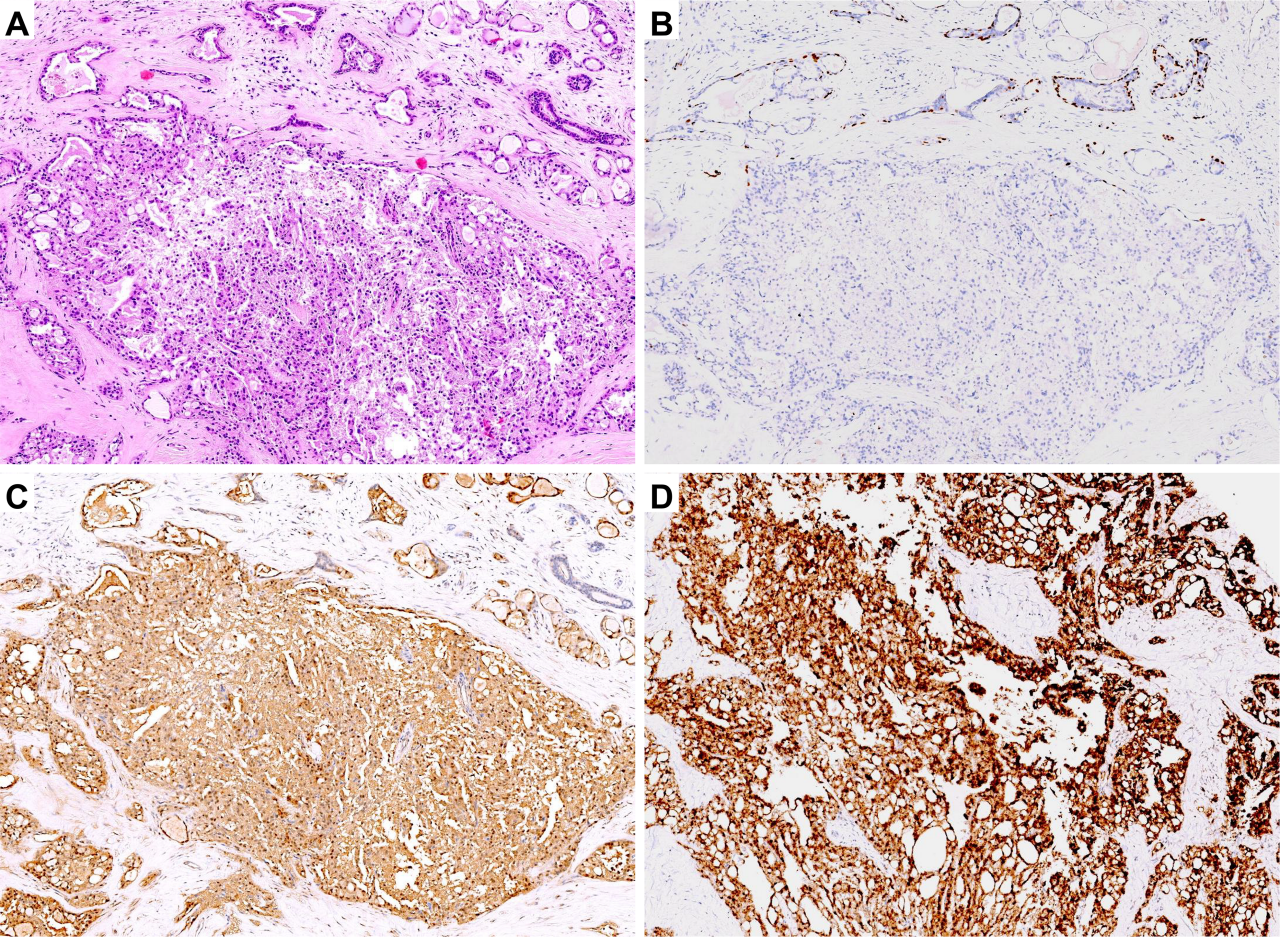
Carcinoma *in situ* (CIS) and invasive components in secretory carcinoma. (A) HE stain showing mixed CIS and invasive carcinoma. (B) p63 immunostaining highlights peripheral myoepithelial cells around ducts, confirming CIS; the absence of myoepithelial cells around adjacent nests indicates invasion. (C) Diffuse S100 positivity in tumour cells, present in both CIS and invasive components. (D) Diffuse nuclear and cytoplasmic positivity for pan‐TRK in tumour cells, present in both CIS and invasive components.

**Figure 3:**
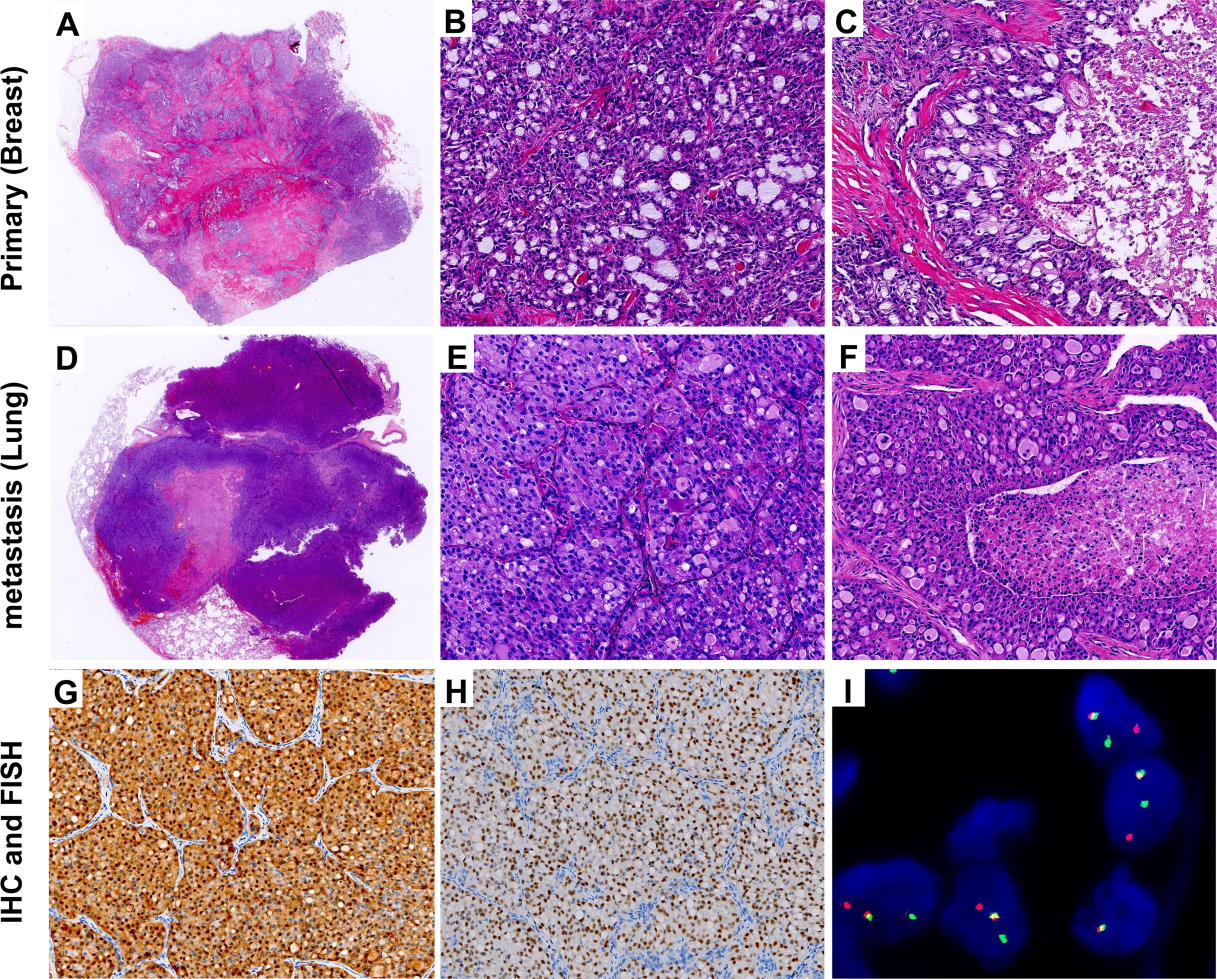
Secretory carcinoma of the breast with pulmonary metastasis. (A–C) Primary breast tumour showing (A) an overview of the tumour, (B) microcystic and solid nests and (C) focal necrosis. (D–F) Pulmonary metastasis showing (D) an overview of the tumour, (E) microcystic, and solid nests and (F) focal necrosis. (G, H) Pulmonary metastasis, immunohistochemistry showing (G) diffuse strong S100 and (H) pan‐TRK positivity. (I) Pulmonary metastasis, FISH analysis demonstrating *NTRK3* gene rearrangement (positive).

Grossly, 25 tumours appeared as well‐circumscribed, round to oval nodules, whereas 4 were ill‐defined and stellate. Tumour size ranged from 0.5 to 4.5 cm (mean, 1.5 cm). Cut surfaces were gray‐white and of firm to moderately firm texture. Microscopically, most tumours exhibited pushing borders with ill‐defined peripheries. The neoplastic cells were arranged predominantly in microcystic (Figure 1A) and tubular structures (Figure 1B), occasionally with solid areas (Figure 1C); Case 15 predominantly exhibited a papillary architecture (Figure 1D). The distribution of architectural combinations was as follows: MC + T in 12/29 (41.4%), MC + T + P in 12/29 (41.4%), MC + T + S in 3/29 (10.3%), MC + P in 1/29 (3.4%), and MC + S in 1/29 (3.4%) (MC, microcystic; T, tubular; S, solid; P, papillary) (Table 1).The cells contained eosinophilic granular or vacuolated cytoplasm, with variable intra‐ and extracellular eosinophilic or basophilic secretions. Stromal hyalinisation was frequent (Figure 1E), and myxoid change was occasionally observed (Figure 1F). Nuclear grade was predominantly grade 1, with a few cases showing intermediate features (grade 1–2). Only the metastatic case (Case 11) demonstrated a higher nuclear grade (grade 2–3) with increased cytologic atypia and brisk mitotic activity (6/10 high‐power fields, HPF), whereas all other tumours showed rare mitoses (0–1/10 HPF). Foci of carcinoma *in situ* (CIS) were present in many tumours, highlighted by p63 immunostaining at the periphery (Figure 2A, B). In the only case with distant metastasis (Case 11), both the primary breast lesion (Figure 3A, B) and lung metastasis (Figure 3D, E) showed predominantly solid growth with only focal microcystic structures. This case displayed increased cytologic atypia, brisk mitotic activity, and multifocal necrosis (Figure 3C, F).

### Immunohistochemistry and FISH

All tumours demonstrated diffuse nuclear and cytoplasmic positivity for S100 (29/29) and pan‐TRK (29/29) (Table 1, Figure 2C, D). Notably, the metastatic case also showed strong diffuse expression of S100 and pan‐TRK in both the primary tumour and the pulmonary metastasis (Figure 3G, H). ER and/or PR expression was generally low, ranging from 2% to 30% of tumour cells (weak to moderate staining), with 13 cases negative and 16 cases showing focal to moderate positivity. All tumours were HER2 negative or equivocal (0 to 1+), with no evidence of HER2 amplification. The Ki‐67 proliferation index was consistently low across the cohort, ranging from 3% to 20% (mean, 7%).

Seventeen cases underwent fluorescence *in situ* hybridisation (FISH), all of which were positive, including 14 with *ETV6‐NTRK3* fusion and 3 with *NTRK3* break‐apart signals. Notably, the metastatic case (Case 11) was confirmed by both FISH and next‐generation sequencing (NGS) (Figure 3I).

### Molecular findings

Next‐generation sequencing (NGS) was performed on the metastatic case (Case 11). RNA sequencing (RNA‐seq) confirmed the canonical *ETV6‐NTRK3* fusion transcript. Targeted DNA sequencing (DNA‐seq) additionally identified several somatic variants of uncertain significance (VUS), including *RANBP2* (p.S1843R), *NUP107* (p.K382Q), *NCOR1* (p.A1947V) (all missense) and *PREX2* (p.G606G) (synonymous). The biological and clinical relevance of these alterations remains unclear.

### Treatment and follow‐up

All 29 patients underwent surgery, including breast‐conserving surgery (*n* = 14, 1 with sentinel lymph node biopsy), simple mastectomy (*n* = 10, 7 with sentinel lymph node biopsy), and modified radical mastectomy (*n* = 5). Fourteen patients received adjuvant chemotherapy, whereas 15 cases did not. Follow‐up information was available for all patients, ranging 6–135 months (mean 66 months; median 76 months) (Table 1).

One patient (Case 11) had axillary lymph node metastasis at diagnosis (3/12 lymph nodes positive), developed pulmonary metastasis 1 year after modified radical mastectomy and adjuvant chemotherapy, and subsequently underwent lung resection. At 88 months of follow‐up, this patient was alive but with recurrent pulmonary metastases. The remaining 28 patients were free of lymph node metastasis at initial diagnosis, remained without recurrence or distant metastasis throughout follow‐up, and all were alive at the last contact.

## Discussion

Secretory breast carcinoma (SBC) is a rare subtype of invasive breast carcinoma, first described by McDivitt and Stewart in 1966 in children, and initially designated as ‘juvenile breast carcinoma’ [1]. Subsequent studies have demonstrated that SBC can occur across all age groups, including adults and male patients [2, 16, 17]. Its incidence among invasive breast carcinomas is estimated to be less than 0.15% [16, 18, 19]. Most of the published literature consists of case reports, small series, or retrospective database analyses, and systematic studies with larger cohorts focusing on the clinicopathologic, immunophenotypic, and molecular features of SBC remain limited [6, 20]. The present single‐centre series of 29 cases from our institution represents one of the largest single‐centre cohorts reported in China to date, aiming to further characterise its clinicopathologic, immunohistochemical, and molecular features, and to explore diagnostic pitfalls and prognostic indicators.

In our cohort, the vast majority of patients were female, with only one male, and the age distribution (12–63 years) was consistent with prior reports [6, 16]. Clinically, SBC often presented as a painless, well‐circumscribed parenchymal mass, with ultrasonography typically showing hypoechoic or mixed echogenicity mimicking benign breast lesions and potentially leading to misdiagnosis [21]. Notably, one case arose in axillary accessory breast tissue, indicating that SBC can also occur in ectopic breast tissue.

Histologically, SBC is usually a low‐grade invasive carcinoma. The characteristic features include microcystic, tubular, and solid nests, occasionally with papillary architecture, although usually admixed with microcystic and tubular patterns. One of our cases exhibited predominantly papillary structures, a finding that has also been documented in rare reports [22, 23]. Tumour cells typically show eosinophilic or vacuolated cytoplasm, with intra‐ and extracellular eosinophilic or basophilic secretions. These secretions are positive for PAS and PAS‐diastase and partly positive for Alcian blue, indicating the presence of acidic mucopolysaccharides or glycoproteins. Hyalinised stroma and associated carcinoma *in situ* (CIS) are common, whereas nuclear atypia is generally mild [2, 3, 6]. Most of our cases showed these classic features. However, in one patient who developed distant metastasis, the primary breast tumour, lymph node metastasis, and pulmonary metastasis were predominantly solid, with only focal microcystic structures. This case also showed increased cytological atypia, frequent mitoses, and multifocal necrosis. Such morphology is uncommon in SBC and has been reported only rarely in metastatic cases [4, 8, 9]. These findings suggest that a solid growth pattern with increased mitotic activity and necrosis may be associated with aggressive biological behaviour, warranting particular attention.

Immunophenotypically, all 29 cases demonstrated strong, diffuse expression of S100 and pan‐TRK, supporting their diagnostic utility as sensitive and specific markers for SBC [15]. Most studies report SBC as triple‐negative (ER−/PR−/HER2−), often expressing basal‐like markers such as CK5/6 and EGFR, leading to its classification as a special subtype of triple‐negative breast carcinoma [3, 4, 24, 25, 26, 27]. In our cohort, however, 16 cases (55%) showed weak to moderate ER and/or PR positivity (2–30% of tumour cells), consistent with early reports and later cohort studies [6, 16, 20]. None of the cases demonstrated *HER2* amplification or overexpression, although approximately one‐third showed HER2‐low expression (1+). The Ki‐67 proliferation index was low (3–20%, mean 7%), in line with the indolent proliferative activity of SBC. Overall, although SBC is most often triple‐negative, hormone receptor expression does not preclude the diagnosis and should be interpreted in conjunction with morphology and specific markers. Diffuse S100 and pan‐TRK positivity remain highly informative clues.

At the molecular level, the hallmark alteration of SBC is the t(12;15)(p13;q25) translocation, resulting in the *ETV6‐NTRK3* fusion gene [4]. This was first identified in SBC by Tognon *et al* in 2002 [5], and subsequent studies have reported its presence in approximately 90% of cases [5, 6, 28]. The resultant chimeric tyrosine kinase constitutively activates the Ras‐MAPK and PI3K‐AKT pathways, driving tumourigenesis [29]. In our study, 17 cases were tested by FISH, of which 14 showed *ETV6‐NTRK3* fusion and 3 showed *NTRK*3 break‐apart signals. Importantly, pan‐TRK immunohistochemistry proved to be a highly sensitive surrogate for *NTRK* fusions in this context, supporting its use as a rapid screening tool in routine practice.

In our cohort, RNA sequencing of the single metastatic case (Case 11) confirmed the canonical *ETV6‐NTRK3* fusion transcript. Concomitant variants of uncertain significance (VUS) were also detected – RANBP2 (p.S1843R), *NUP107* (p.K382Q), and *NCOR1* (p.A1947V) as missense changes, and a synonymous *PREX2* (p.G606G) variant – none of which currently have an established role in SBC. Given the characteristically low mutational burden of SBC, these alterations are best regarded as putative passenger events pending functional validation. Nevertheless, their co‐occurrence in the solid‐predominant, metastatic tumour raises the hypothesis that additional cooperating events might contribute to aggressive behaviour in a small subset of cases.

While some laboratories employ *ETV6* break‐apart probes as surrogates for *NTRK* fusion screening, caution is warranted because *ETV6* rearrangements are not specific to SBC. For example, *BCL2L14‐ETV6* fusions have been reported in high‐grade invasive ductal carcinomas [30]. Thus, in morphologically atypical cases, *ETV6* rearrangement alone should not be overinterpreted as diagnostic of SBC. Apart from *NTRK* fusions, rare recurrent alterations such as *TERT* promoter mutations have been reported in metastatic or recurrent SBC [7, 31]. Nevertheless, the overall mutational burden of SBC is low, with few recurrent mutations or copy‐number alterations [32, 33]. Moreover, different exon breakpoints in *ETV6‐NTRK3* fusions give rise to multiple transcript variants [5, 34], which may share overlapping functions yet exhibit biological differences [35]. Further studies are needed to delineate their mechanistic diversity and therapeutic implications.

The differential diagnosis of SBC includes several morphologically overlapping entities. Its microcystic structures, eosinophilic cytoplasm, and secretory material may resemble apocrine carcinoma, cystic hypersecretory carcinoma, adenoid cystic carcinoma, and lactating adenoma. Cases with predominant papillary architecture may mimic papillary neoplasms. In routine diagnosis, accurate distinction is usually achievable by combining morphology with immunohistochemistry and, when indicated, molecular testing. Of note, the sclerotic stroma often seen in SBC may be mistaken for sclerosing adenosis, and awareness of this pitfall is important to avoid misdiagnosis.

Given the generally indolent behaviour of SBC, surgery remains the mainstay of treatment. In our cohort, all patients underwent surgical resection, including breast‐conserving surgery, simple mastectomy with sentinel lymph node biopsy, or modified radical mastectomy, tailored to tumour size and clinical assessment. No standardised surgical guidelines currently exist, but local excision or mastectomy with sentinel lymph node biopsy is generally considered reasonable for tumours smaller than 2 cm [3, 24]. There are no specific recommendations for adjuvant therapy in SBC. Because most SBCs are triple‐negative, adjuvant chemotherapy is often administered following protocols for triple‐negative breast cancer, typically anthracycline‐ or taxane‐based. However, the chemosensitivity of SBC remains uncertain, and some patients relapse despite multiple courses of chemotherapy [9, 34]. In our metastatic case, recurrence occurred within 1 year despite adjuvant chemotherapy, underscoring its limited efficacy. Moreover, late recurrences have been reported, sometimes up to 20 years after initial diagnosis [36], highlighting the need for long‐term surveillance. With the approval of *NTRK* inhibitors such as larotrectinib and entrectinib, detection of *ETV6‐NTRK3* fusions not only provides a diagnostic hallmark but also offers a potential therapeutic target in advanced or refractory cases [37, 38].

In summary, this cohort of 29 SBC cases represents one of the largest single‐institution series in China. Although the majority of cases pursue an indolent course with favourable outcomes, our findings emphasise the morphologic and biological heterogeneity of this entity. Notably, tumours exhibiting predominantly solid growth, increased cytological atypia, high mitotic activity, and necrosis may demonstrate more aggressive clinical behaviour. A comprehensive diagnosis requires the integration of morphology, immunohistochemistry, and molecular testing, with particular emphasis on *NTRK* analysis. *RNA‐seq and DNA‐seq provide complementary information, with RNA‐seq confirming canonical fusions and DNA‐seq surveying for additional genetic alterations such as VUS, which may contribute to aggressive behaviour in rare cases*. Recognising the morphological spectrum and molecular characteristics of SBC is essential to improve diagnostic accuracy and to inform individualised patient management, *particularly in advanced or refractory disease*.

## Author contributions statement

All authors meet the authorship requirements. HR participated in the design of the study, drafted the manuscript, and performed the experiments. WT, JM, XC, HH, NW, YL, MY, LZ, HL analysed the data and prepared the figures. All authors read and approved the final manuscript.

## Data Availability

The data that support the findings of this study are available from the corresponding author upon reasonable request.
